# Leveling Method of Working Platform Based on PZT Electromechanical Coupling Effect

**DOI:** 10.3390/mi16070796

**Published:** 2025-07-08

**Authors:** Aiqun Xu, Jianhui Yuan, Jinxuan Gao

**Affiliations:** School of Intelligent Manufacturing and Energy Engineering, Zhejiang University of Science and Technology, 318 Liu He Road, Hangzhou 310023, China; g3250926715@163.com

**Keywords:** leveling, piezoelectric material, electromechanical coupling effect

## Abstract

Lead zirconate titanate (PZT) piezoelectric ceramics are widely used functional materials due to their strong and stable piezoelectric properties. A leveling method based on lead zirconate titanate piezoelectric ceramics is proposed for the high level of accuracy required in microelectromechanical fields such as aerospace, industrial robotics, biomedical, and photolithography. A leveling mechanism consisting of core components such as piezoelectric ceramic actuators and sensors is designed. The closed-loop leveling of the working platform is performed using the electromechanical coupling effect of the PZT piezoelectric material. Combined with the theory of the dielectric inverse piezoelectric effect in electric fields, a simulation is used to analyze the four force and deformation cases generated by the drive legs when the load is attached at different positions of the working platform, and the leveling is realized by applying the drive voltage to generate micro-motion displacement. Simulation and calculation results show that the leveling method can reduce the tilt angle of the working platform by 60% when the driving voltage is in the range of 10~150 V. The feasibility of the leveling method and the uniformity of the theoretical calculation and simulation are verified.

## 1. Introduction

It is especially important to keep the working platform level during the transportation, processing, and assembly of parts. With the development of microelectromechanical systems, most mechanical devices have higher and higher requirements for leveling [1,2], and the working platform of many mechanical devices will be tilted slightly under force, which will seriously affect the quality and economic efficiency of the final product due to the accumulation of various errors, and then it has to be leveled.

In the field of leveling methods, the variable detected by the traditional position feedback leveling method is the displacement of the platform, and since the displacement is the result of the force, the displacement of the platform has an obvious hysteresis compared with the force, and in the ultra-precision grinding process of silicon wafers, the feedback hysteresis will lead to a decrease in the precision of the control of the shape of the silicon wafer face, so the traditional position feedback leveling method is not suitable for the ultra-precision grinding process of silicon wafers. Therefore, the traditional position feedback leveling method is not suitable for dynamic adjustment in the wafer ultra-precision grinding process, which hinders the further improvement of processing accuracy. A method of platform leveling based on a hydraulic control system with a multi-point support structure was earlier proposed by Southeast University, which is suitable for heavy load occasions but not for microsystems and in precision machinery, and the hydraulic system is greatly affected by temperature, so the application area is limited, and the realization cost is high [3]. A servo motor-controlled four-pivot support platform is used, and finally an inclination sensor is used to detect whether it is level or not. In the leveling process, the four legs are prone to inconsistent force, which causes the servo motor to be overloaded [4].

The above leveling method has a limited horizontal adjustment accuracy, which cannot meet the requirements for the processing and assembly of precision parts in terms of the horizontal accuracy of the working platform, and most of them use a four-point support structure, which can easily produce a “false leg” phenomenon due to uneven force [5]. The use of an equilateral three-leg structure, compared with a four-point support structure, has the advantages of good stability and a high reliability of support, avoiding the phenomenon of uneven force on the legs due to “false legs”, and the application is more widespread. And previous research on piezoelectric ceramics has focused on the properties of the material itself and energy storage [6]. In this regard, a leveling method based on the inherent piezoelectric strain property of PZT functional materials is proposed. The adjustment of the working platform is based on the micro-motion displacement of the material itself, which avoids the error caused by hydraulic and motor media, and it has a clear control logic, fast response speed, and high adjustment accuracy, etc., characteristics [7,8].

## 2. Leveling Principle

In the absence of an external electric field, the electric domains on the surface of the piezoelectric ceramic cancel each other due to the internal polarization, and after polarization, the internal domains spontaneously polarize in the direction of the applied electric field, which shows the piezoelectric effect. The inverse piezoelectric effect of piezoelectric ceramics is based on the principle that the piezoelectric ceramic is polarized by the electric field and deformed by the electric field force [9,10,11].

**Figure 1:**
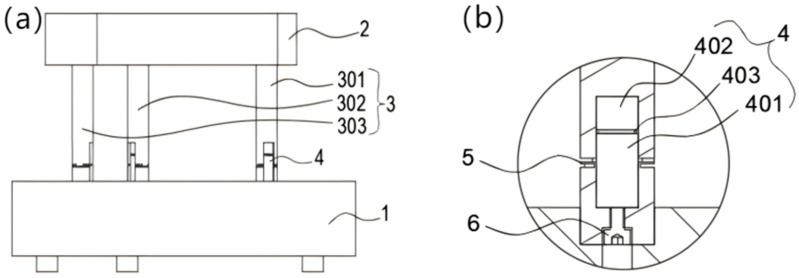
Overall structure (**a**) and partial diagram (**b**). 1—base, 2—working platform, 3—drivable legs, 4—piezoelectric ceramic module, 5—flexible hinge, 6—prefixing screws, 301—first drivable leg, 302—second drivable leg, 303—third drivable leg, 401—piezoelectric ceramic driver, 402—piezoelectric ceramic sensor, 403—epoxy resin.

The overall structure of the leveling mechanism is shown in Figure 1a, and Figure 1b shows a partial view of the piezoelectric ceramic module (4). The working platform (2) is used to carry the working load and has an equilateral triangular structure in the top view. The base (1) and the working platform are connected by three actuatable legs (3) via pretensioning screws (6), which are distributed in an equilateral triangle. At the piezoelectric ceramic module (4), the piezoelectric ceramic actuator (401), the sensor (402), and the electrical insulation material epoxy (403) are arranged sequentially in the vertical direction, the sensor is used to detect the voltage, and the actuator is used to control the expansion and contraction of the legs. The epoxy resin is used as an insulating binder for the driver and sensor. The flexible hinge (5) provides axial support for the actuatable legs. Because of the axial restraint of the base on the actuatable legs, the displacement of the legs varies along the vertical direction when the working platform is subjected to a working load. By comparing the magnitude relationship of each leg component’s force to apply the driving voltage to control the micro-motion displacement of the legs, the leveling of the working platform is realized.

The magnitude of the micro-motion displacement of the piezoelectric ceramic is controlled by the voltage applied to the functional material of the PZT. The leveling sequence is as follows: working load → force on the legs → deformation of PZT → comparison of the magnitude of each partial force → application of driving voltage → generation of micro-motion displacement → difference in partial force within the accuracy threshold → end of leveling. The leveling process has almost no energy loss and can meet the requirements of high-precision leveling.

### 2.1. Leveling Steps

**Figure 2:**
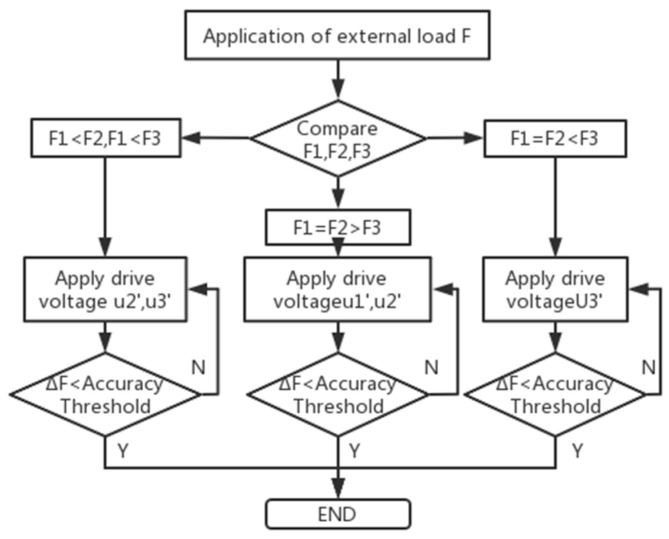
Workflow diagram.

The load F is applied to the working platform, and the three drivable legs are divided into F_1_, F_2_, F_3_, and the corresponding voltage values generated by the piezoelectric ceramic sensors in the piezoelectric ceramic module are U_1_, U_2_, and U_3_. The workflow diagram is shown in Figure 2. According to the inverse piezoelectric effect of piezoelectric ceramics in electric fields [12], the piezoelectric ceramic strain can be obtained from Equation (1). After applying the driving voltage, the relationship between the driving force F_1_, F_2_, F_3_ and the driving voltages U_1_, U_2_, U_3_ generated by the piezoelectric ceramic driver is shown in Equation (2).

(1) $$\varepsilon =\frac{cU}{d}+M\frac{U^{2}}{d^{2}},$$

(2) $$\left.\begin{array}{c} F_{1}=\left(\frac{U_{1}}{d}c+\frac{U_{1}^{2}}{d^{2}}M\right)EA\\ F_{2}=\left(\frac{U_{2}}{d}c+\frac{U_{2}^{2}}{d^{2}}M\right)EA\\ F_{3}=\left(\frac{U_{3}}{d}c+\frac{U_{3}^{2}}{d^{2}}M\right)EA \end{array}\right\},$$

ε: Stretch of piezoelectric ceramic actuator;

*c*: Piezoelectric coefficient of piezoelectric ceramic material;

*U*_1_, *U*_2_, *U*_3_: the driving voltage of each piezoelectric ceramic driver;

*d*: Distance between the single set of poles of the piezoelectric ceramic driver or piezoelectric ceramic sensor;

*M*: Electrostriction coefficient of piezoelectric ceramic material;

*A*: Cross-sectional area of the drivable legs;

*E*: Young’s modulus of PZT material;

*F*_1_, *F*_2_, *F*_3_: Fractional force applied to each leg.

**Figure 3:**
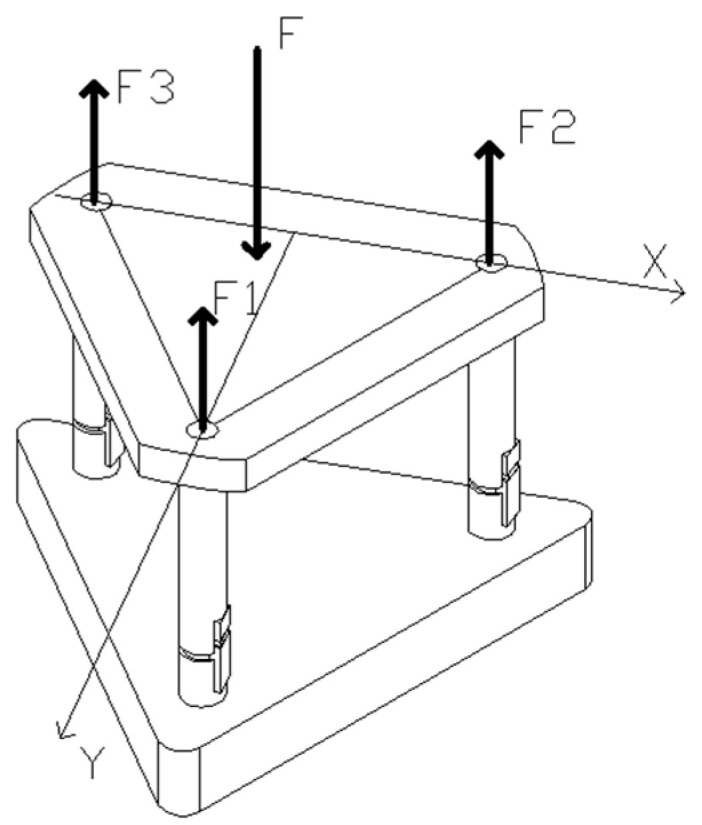
Schematic diagram of the parting forces on each drive leg.

When the working platform is subjected to external force F, the component forces on each leg are F_1_, F_2_, and F_3_, as shown in Figure 3. Where a is one-half of the side length of the equilateral triangle working platform, x and y are the coordinates of the point where the force F acts on the working platform. According to the space parallel force system, the relationship between the component forces of each leg can be found as shown in Equation (3).

(3) $$\left.\begin{array}{c} F=F_{1}+F_{2}+F_{3}\\ F_{2}a-Fx=F_{3}a\\ \sqrt{3}F_{1}a=Fy \end{array}\right\}$$

After finding F_1_, F_2_, and F_3_ according to Equation (3), the force situation of each drivable leg can be divided into four cases, such as F_1_ = F_2_ = F_3_, F_1_ < F_2_ and F_1_ < F_3_, F_1_ = F_2_ < F_3_ and F_1_ = F_2_ > F_3_. In practice, to ensure that the working platform is not unbalanced by the overturning moment, the force point area of the leveling mechanism is distributed in an equilateral triangle.

(1) When F_1_ = F_2_ = F_3_, the working platform is in a horizontal state, and no leveling is needed.
(2) When F_1_ < F_2_ and F_1_ < F_3_, the partition forces on each drivable leg are not the same. From Hooke’s law, we can see that the deformation variables of each drivable leg under the action of F_1_, F_2_, and F_3_ are as follows:
  (4) $$\Delta L_{1}=\frac{F_{1}L}{EA},\Delta L_{2}=\frac{F_{2}L}{EA},\Delta L_{3}=\frac{F_{3}L}{EA},$$
  where ΔLi(i = 1, 2, 3) corresponds to the deformation of each drivable leg; Fi(i = 1, 2, 3) corresponds to the pressure shared by one, two, and three drivable legs, respectively; and L is the length of the drivable leg. In the initial state, the lengths of the drivable legs are equal. At this time, the difference in the deformation of the three drivable legs is
  (5) $$\left.\begin{array}{c} \Delta L_{2}-\Delta L_{1}=\frac{\left(F_{2}-F_{1}\right)L}{EA}\\ \Delta L_{3}-\Delta L_{1}=\frac{\left(F_{3}-F_{1}\right)L}{EA} \end{array}\right\},$$

To restore the platform to a horizontal state at this point, the deformation of the second piezoelectric actuator should be made equal to ΔL_2_ − ΔL_1_ and the deformation of the third piezoelectric actuator equal to ΔL_3_ − ΔL_1_. L_p_ is the length of the piezoelectric ceramic actuator.

(6) $$\left.\begin{array}{c} \Delta L_{2}-\Delta L_{1}=\frac{\left(F_{2}-F_{1}\right)L}{EA}=\left(\frac{U_{2}^{\prime }}{d}c+\frac{U_{2}^{\prime 2}}{d^{2}}M\right)L_{p}\\ \Delta L_{3}-\Delta L_{1}=\frac{\left(F_{3}-F_{1}\right)L}{EA}=\left(\frac{U_{3}^{\prime }}{d}c+\frac{U_{3}^{\prime 2}}{d^{2}}M\right)L_{p} \end{array}\right\},$$

A drive voltage U_2_′ is applied to the piezoelectric ceramic driver at the second drivable leg, and a drive voltage U_3_′ is applied to the piezoelectric ceramic driver at the third drivable leg.

(7) $$\left.\begin{array}{c} U_{2}^{\prime }=\frac{-cd+\sqrt{c^{2}d^{2}+4Md^{2}\frac{\left(F_{2}-F_{1}\right)L}{EAL_{p}}}}{2M}\\ U_{3}^{\prime }=\frac{-cd+\sqrt{c^{2}d^{2}+4Md^{2}\frac{\left(F_{3}-F_{1}\right)L}{EAL_{p}}}}{2M} \end{array}\right\},$$

The incremental force ΔF_1_ in the first drivable leg is detected due to the expansion and contraction of the second and third drivable legs, where ΔU_1_ is the voltage generated by the piezoelectric ceramic sensor on the first drivable leg under the action of ΔF_1_; δ is the accuracy threshold, the value of which is set according to the required leveling accuracy of the actual working platform; if ΔF_1_ < δ, the leveling is finished.

(8) $$\Delta F_{1}=\left(\frac{\Delta U_{1}}{d}c+\frac{\Delta U_{1}^{2}}{d^{2}}M\right)EA,$$

Otherwise the second and third drivable legs of the piezoelectric ceramic driver apply drive voltages modified to U_2_″, U_3_″.

(9) $$U_{2}^{\prime \prime }=U_{2}^{\prime }-\Delta U_{1}k_{1},$$

(10) $$U_{3}^{\prime \prime }=U_{3}^{\prime }-\Delta U_{1}\left(1-k_{1}\right),$$

(11) $$k_{1}=\frac{U_{2}^{\prime }}{U_{2}^{\prime }+U_{3}^{\prime }},$$

When F_1_ = F_2_ < F_3_, a driving voltage is applied to the piezoelectric ceramic driver at the third drivable leg, and an increment in the component force in the first drivable leg due to the expansion and contraction of the third drivable leg is detected.

(12) $$U_{3}^{\prime }=\frac{-cd+\sqrt{c^{2}d^{2}+4Md^{2}\frac{\left(F_{3}-F_{1}\right)L}{EAL_{p}}}}{2M},$$

(13) $$\Delta F_{1}^{\prime }=\left(\frac{\Delta U_{1}^{\prime }}{d}c+\frac{\Delta U_{1}^{\prime 2}}{d^{2}}M\right)EA,$$

According to a comparison of multiple flatness detection methods [13], if ΔF_1_′ < δ, leveling is finished. Otherwise, re-apply the drive voltage U_3_″ to the piezoelectric ceramic driver at the third drivable leg.

(14) $$U_{3}^{\prime \prime }=U_{3}^{\prime }-\Delta U_{1},$$

When F_1_ = F_2_ > F_3_, it is necessary to apply an equal voltage U_1_′ to both legs 1 and 2. The resulting incremental force of the two legs 1 and 2 is obtained. If ΔF_1_′ > δ, re-apply the driving voltage U_1_″.

(15) $$U_{1}^{\prime }=U_{2}^{\prime }=\frac{-cd+\sqrt{c^{2}d^{2}+4Md^{2}\frac{\left(F_{1}-F_{3}\right)L}{EAL_{p}}}}{2M},$$

(16) $$\Delta F_{1}^{\prime }=\Delta F_{2}^{\prime }=\left(\frac{\Delta U_{1}^{\prime }}{d}c+\frac{\Delta U_{1}^{\prime 2}}{d^{2}}M\right)EA,$$

(17) $$U_{1}^{\prime \prime }=U_{2}^{\prime \prime }=U_{1}^{\prime }-\Delta U_{3},$$

### 2.2. Analysis of Tilt Angle of Working Platform

After the leveling step is completed, the levelness analysis is to be carried out. Here the size of the tilt angle of the platform is used to measure the level of the working platform. The tilt angle is the angle between the plane of the working platform after tilting and the reference plane, so as to judge whether the levelness of the working platform meets the accuracy requirements of the working condition [14,15,16]. After the working platform is tilted by external force, the level of the working platform is analyzed by analyzing the relationship between the tilt angle and the leg shape variables.

**Figure 4:**
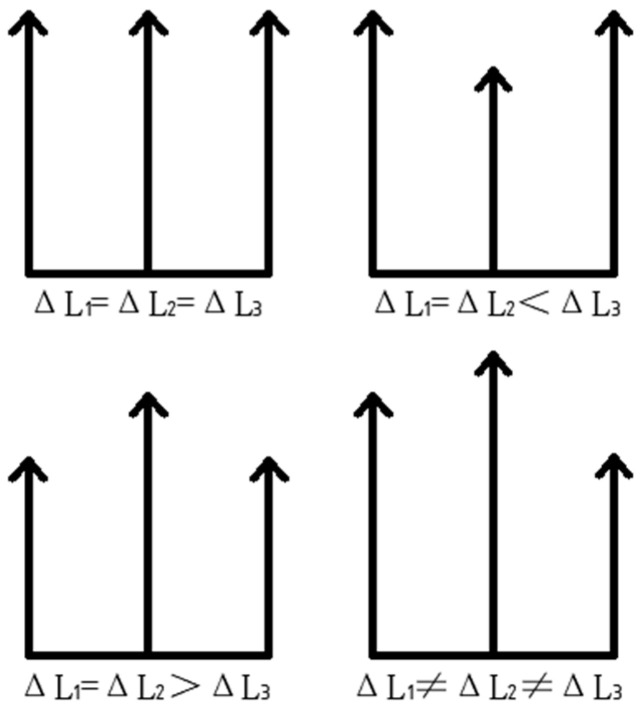
Deformation of each leg under force.

From the analysis of the above leveling steps, it is known that there are four cases of force deformation for each leg when the working platform is subjected to force, as shown in Figure 4 below.

**Figure 5:**
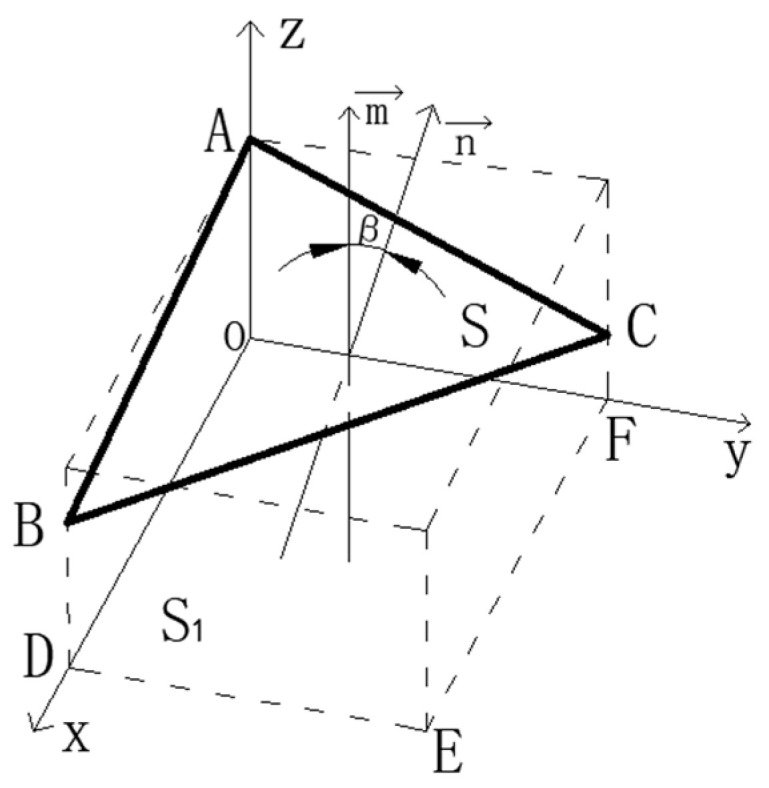
Schematic diagram of tilt angle.

When the deformation of the legs is ΔL_1_ ≠ ΔL_2_ ≠ ΔL_3_, as shown in Figure 5, since the deformation of each leg is different, the working platform can be tilted in any direction in the three-dimensional space, and the calculation of the tilt angle is analyzed by the vector method.

In the coordinate system A(0,0,z1), B(x,0,z2), C(0,y,z3), D(x,0,0), E(x,y,0), F(0,y,0), let the platform ABC force A point leg force be the smallest, the C point leg force be the largest, then the deformation between the legs for z_1_-z_3_ = ΔL_3_-ΔL_1_, z_1_-z_2_ = ΔL_2_-ΔL_1_, and then with the above Formula (5) substitution, the normal vector of the work platform ABC $\vec{n}$ = $\left(1,\frac{x\left(F_{3}-F_{1}\right)}{y\left(F_{2}-F_{1}\right)},\frac{xEA}{\left(F_{2}-F_{1}\right)L}\right)$, the horizontal reference surface S1 normal vector $\vec{m}$ = (0,0,1), find the cosβ of the angle between the surface S and S1; at this time β is the angle of inclination, that is

(18) $$\begin{array}{r} \cos\beta =\frac{\vec{n}\times \vec{m}}{\left|\vec{n}\right|\left|\vec{m}\right|}=\frac{x_{1}x_{2}+y_{1}y_{2}+z_{1}z_{2}}{\sqrt{x_{1}^{2}+y_{1}^{2}+z_{1}^{2}}\sqrt{x_{2}^{2}+y_{2}^{2}+z_{2}^{2}}}\\ =\frac{xEA}{\sqrt{{\left[\left(F_{2}-F_{1}\right)L\right]}^{2}+{\left[\left(F_{3}-F_{1}\right)\frac{x}{y}L\right]}^{2}+{\left(xEA\right)}^{2}}} \end{array}$$

**Figure 6:**
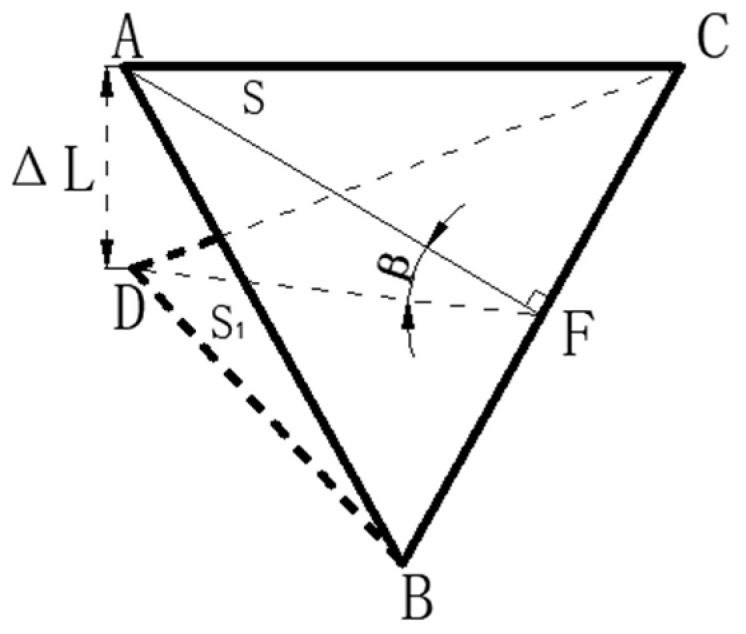
Geometric relationship between working platform surface and inclined surface.

When the deformation of the legs is ΔL_1_ = ΔL_2_ < ΔL_3_ or ΔL_1_ = ΔL_2_ < ΔL_3_, as shown in Figure 6, since there are always two legs with equal deformation variables, the working platform only tilts in the same direction, and the trigonometric method can be used directly in analyzing the tilt angle.

The tilt angle between the working platform ABC and the pressurized inclined plane DCB is still β (the two planes are represented by S and S1, respectively), and ΔL is the pressurized deformation variable of the two planes in the axial direction. And AF ⊥ BC, because the side length of the working platform is 2a, so AF = a.

From Figure 6, the mathematical relationship between the leg shape variables and the tilt angle of the working platform is

(19) $$\sin\beta =\frac{\Delta L}{AF},$$

Since → 0, the above equation can be simplified as

sinβ≈β

When the working platform is under force, the deformation variables of each leg are shown in Equation (4). Substituting Equation (4) into Equation (18), we can get the tilt angle of each leg due to the deformation variables as follows:

(20) $$\beta =\frac{\Delta L_{1}}{AF}=\frac{\sqrt{3}}{3a}\frac{F_{1}L}{EA},$$

When the working platform is subjected to the force to produce the tilt angle, according to the above-mentioned leveling steps, the difference in the leg shape variables of Equation (6) is substituted into Equation (18) to obtain the theoretical tilt angle of leveling.

(21) $$\beta^{\prime }=\frac{\Delta L_{2}-\Delta L_{1}}{\sqrt{3}a}=\frac{\sqrt{3}}{3}\left(\frac{U_{2}}{d}c+\frac{U_{2}^{\prime 2}}{d^{2}}M\right)\frac{L_{p}}{a},$$

After the leveling step is completed, when verifying the levelness of the working platform, the tilt angle can be obtained by substituting F into Equation (19) as follows:

(22) $$\beta^{\prime }=\frac{\sqrt{3}}{3}\frac{\Delta F}{EA}\frac{L_{p}}{a},$$

And since ΔF < δ, substituting Equation (13) into Equation (22) above, the tilt angle and the accuracy threshold satisfy the following relationship:

$$1.59\;\times \;10^{7}\beta '=\Delta F<\delta$$

## 3. ANSYS Simulation

### 3.1. Pre-Simulation Processing

The piezoelectric sheet is selected as PZT-4 piezoelectric material with density ρ = 7450 kg/m^3^, Poisson’s ratio v = 0.269, Young’s modulus E = 1.06 × 10^11^ Pa. The flexibility matrix S(10^10^ N/m^2^), piezoelectric strain constant matrix d(C/N), and dielectric constant matrix ε(F/m) are selected for the leg structure.

$$S=\left[\begin{array}{cccccc} 12.9 & -5.2 & -5.7 & 0 & 0 & 0\\ -5.2 & 12.9 & -5.7 & 0 & 0 & 0\\ -5.7 & -5.7 & 15.5 & 0 & 0 & 0\\ 0 & 0 & 0 & 36.2 & 0 & 0\\ 0 & 0 & 0 & 0 & 39 & 0\\ 0 & 0 & 0 & 0 & 0 & 39 \end{array}\right],d=\left[\begin{array}{ccc} 0 & 0 & -123\\ 0 & 0 & -123\\ 0 & 0 & 270\\ 0 & 0 & 0\\ 0 & 420 & 0\\ 420 & 0 & 0 \end{array}\right],\varepsilon =\left[\begin{array}{ccc} 882.7 & 0 & 0\\ 0 & 882.7 & 0\\ 0 & 0 & 742.6 \end{array}\right]$$

**Figure 7:**
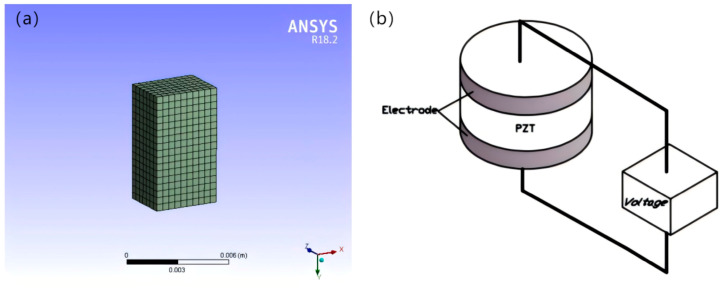
Schematic diagram of piezoelectric sheet meshing (**a**), schematic diagram of piezoelectric drive module (**b**).

In the project, the leveling mechanism is meshed first. The meshing method determines the accuracy of the simulation results, and Figure 7a shows the effect of meshing the piezoelectric ceramic. 

### 3.2. Loading and Solving

When carrying out the piezoelectric solution for the drive voltage, the PZT material layer has multiple piezoelectric ceramic wafers stacked with insulating material electrodes on the top and bottom, as shown in Figure 7b. The piezoelectric solution is performed by the driver applying voltage to the upper and lower electrode layers along the axial direction.

#### 3.2.1. Simulation of Force Distribution on the Legs

**Figure 8:**
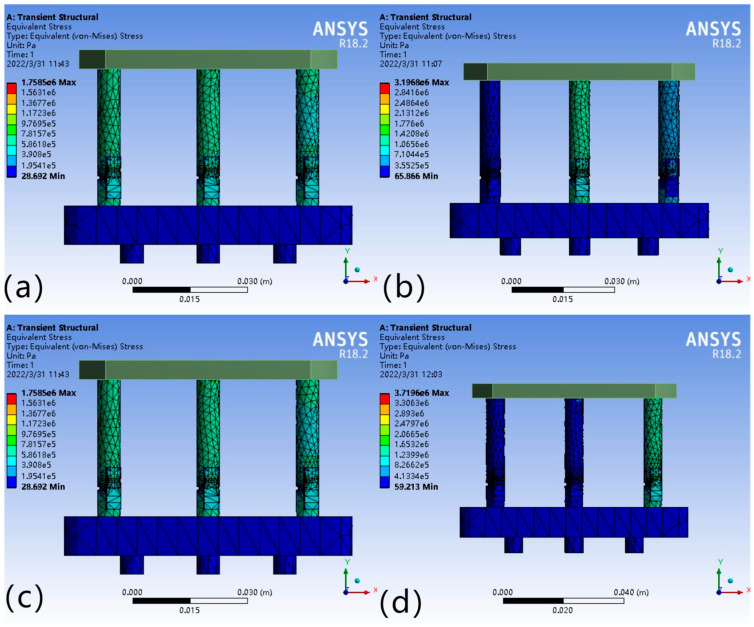
F_1_ = F_2_ = F_3_ (**a**); F_1_ = F_2_ < F_3_ (**b**); F_1_ = F_2_ > F_3_ (**c**); F_1_ < F_2_,F_1_ < F_3_ (**d**).

In the leveling analysis, in order to clarify which leg should be applied voltage, it is necessary to understand the force situation of each leg. When analyzing the force situation of the legs, the simulated load of the working platform is defined as F = 50 N. When the external load point changes, the force condition of the legs will change; so the force point is set in the center point of the working platform, the center line, and other special areas, and the deformation and force of each drivable leg are the following four cases (Figure 8).

**Figure 9:**
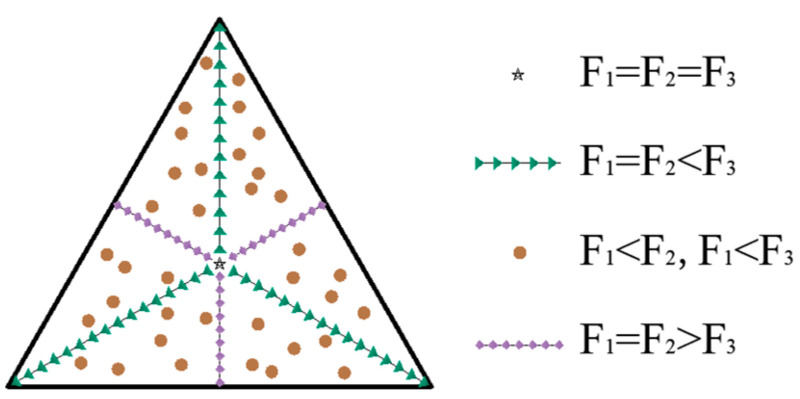
Equilateral triangular working platform force distribution.

From the results in Figure 8a, when the force point is at the center point, the force on each leg is equal, i.e., F_1_ = F_2_ = F_3_; when the force point is distributed on the center line, the force on two of the legs is equal, as shown in Figure 8b,c; Figure 8d indicates that when the force point is located in other areas, the force on each leg is not equal, as expressed by F_1_ < F_2_ and F_1_ < F_3_. The relationship between the force area of the working platform and the force distribution of each leg is shown in Figure 9.

#### 3.2.2. Simulation of Leg Force Deformation

**Figure 10:**
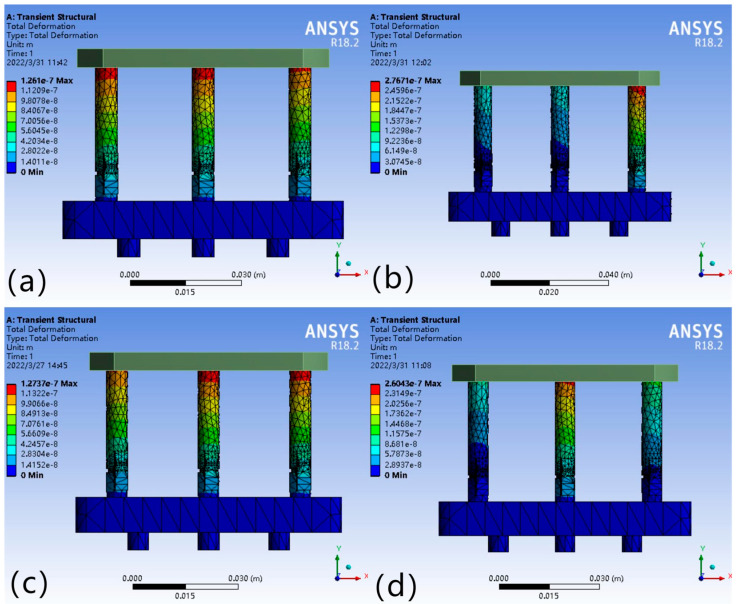
(**a**) ΔL_1_ = ΔL_2_ = ΔL_3_; (**b**) ΔL_1_ = ΔL_2_ < ΔL_3_; (**c**) ΔL_1_ = ΔL_2_ > ΔL_3_; (**d**) ΔL_1_ < ΔL_2_, ΔL_1_ < ΔL_3_.

After analyzing the force on the legs, it is also necessary to analyze the deformation due to the force in order to analyze the required micro-displacement compensation. After obtaining the deformation of each leg through simulation, the drive module must be compensated for by applying a drive voltage to produce a displacement of equal magnitude. Figure 10 show the deformation of the legs under the four force cases. When the force point is located in different areas of the working platform, the deformation of each leg varies positively with the magnitude of the partial force.

As shown in Figure 10a, the deformation variables of each leg are equal, i.e., ΔL_1_ = ΔL_2_ = ΔL_3_; Figure 10b,c show that the deformation variables are ΔL_1_ = ΔL_2_ < ΔL_3_ and ΔL_1_ = ΔL_2_ > ΔL_3_, where the deformation variables of two legs, ΔL_1_ and ΔL_2_, range from −3.07 × 10^−8^ to −9.22 × 10^−8^ m and −1.42 × 10^−8^~−1.27 × 10^−7^ m; ΔL_3_ ranges from −3.07 × 10^−8^ to −2.77 × 10^−7^ m and −1.42 × 10^−8^ to −1.13 × 10^−7^ m, respectively; Figure 10d indicates that the relationship between the deformation variables of each leg is ΔL_1_ < ΔL_2_ and ΔL_1_ < ΔL_3_, where the deformation ranges are −2.89 × 10^−8^ to −8.68 × 10^−8^, −2.89 × 10^−8^~−1.16 × 10^−7^, −2.89 × 10^−8^~−2.60 × 10^−7^ m.

## 4. Analysis of Leveling Results

When analyzing the leveling results of the working platform, the theoretical calculation and simulation analysis of the platform tilt angle are carried out from two perspectives, taking the force situation of the legs with F_1_ = F_2_ < F_3_ as an example.

### 4.1. Theoretical Analysis of Tilt Angle

**Figure 11:**
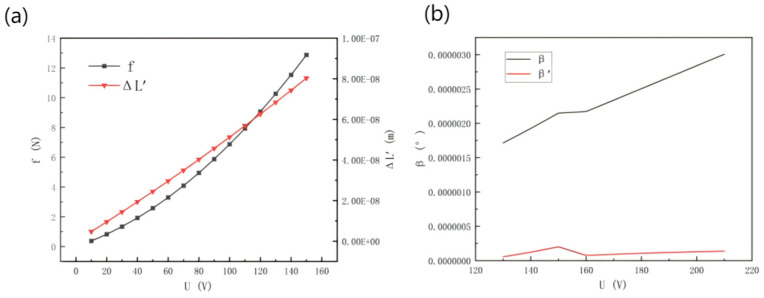
(**a**) Piezoelectric ceramic drive voltage theoretical force and deformation relationship. (**b**) Tilt angle change before and after leveling.

Figure 11a shows the theoretical deformation of the piezoelectric ceramic and the driving force relationship curve obtained when substituting the above Equations (2) and (6) when the driving voltage is in the range of 10~150 v. From the driving force curve, it can be seen that the driving force f generated by applying the driving voltage to the piezoelectric ceramic driver is in the range of 0–13 N, which is within the same magnitude as the partial force F applied to the working platform leg; from the piezoelectric ceramic deformation curve, it can be seen that the displacement generated by applying the voltage is in the range of +10^−7^~10^−8^ m. Compared with the simulation results of the above-mentioned force deformation, it can be verified that the deformation of the leg under force and the piezoelectric ceramic micro-displacement are in the same range. The unification of the micro-displacement produced by the ceramic.

From the theoretical calculation, combined with the above Equations (20) and (21), Figure 11b shows the change curve of tilt angle of the working platform before and after leveling. The tilt angle generated when the platform is subjected to external force is β, and the tilt angle after leveling is β’. The maximum tilt angle of the working platform when subjected to external force is 2.15 × 10^−6^°, and the tilt angle is reduced by 1.95 × 10^−6^° after applying driving voltage. Theoretical calculation shows that before and after leveling, the tilt angle of the working platform can be reduced to 90%.

### 4.2. Tilt Angle Simulation Analysis

In the ANSYS (version 18) piezoelectric coupling simulation analysis, the piezoelectric module is modeled according to the specific working conditions, the length of the piezoelectric ceramic driver itself is 7 mm, and the driving voltage range is 10–150 V. When the stress acts on the cross-section of the drivable leg, compressive deformation is produced in the axial direction. When a voltage is applied at the ends of the piezoelectric ceramic, a corresponding micro-dynamic displacement is produced in the axial direction of the actuatable leg. When an external force of 50 N acts on the centerline of the platform, taking the simulated stress value of 6 × 10^5^ Pa at the piezoelectric ceramic, the cross-sectional area of the leg is S = 25.13 × 10^−6^ m^2^, and through the calculation of the above working principle, a leg force F = 15.078 N can be obtained by substituting the above Equation (2) to obtain the theoretical value of the drive supply voltage of the leg’s piezoelectric ceramic driver under the above working condition, which is about 43 V, and the theoretical deformation variable is −1.038 × 10^−7^ m.

**Figure 12:**
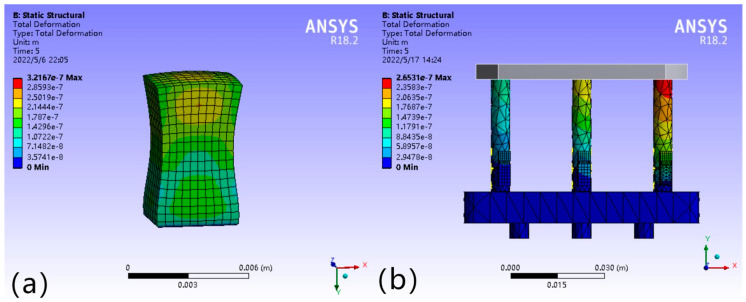
(**a**) Simulation results of piezoelectric ceramic micro-displacement. (**b**) Deformation of each leg after leveling by force difference.

Figure 12a shows the simulation results of the micro-displacement at a voltage value of 43 V, and the simulation value of the micro-displacement of the leg can be basically located in the range of +1.072 × 10^−7^~ + 1.787 × 10^−7^ m. Compared with the simulation results of leg deformation variables in Figure 10b–d, the simulation results of piezoelectric ceramic micro-displacement are within the same magnitude as the theoretical deformation variables when subjected to the leg parting force, the theoretical deformation variables ΔL = −1.038 × 10^−7^ m, and the error value of simulation results is 0.034 × 10^−7^ m, and the error fluctuation is 3.276%, which is within a reasonable range.

According to the simulation results in Figure 10b above (ΔL_1_ = ΔL_2_ < ΔL_3_), when the working platform is subjected to external force, the deformation threshold of the legs subjected to the differential force ΔF ΔL = 4.65 × 10^−8^ m. Figure 12b below shows the distribution of deformation of each leg subjected to the differential force after the driving voltage is applied to the piezoelectric ceramic driver of the F_3_ leg, and the theoretical calculation can be obtained as ΔL The theoretical calculation shows that ΔL’ = −2.94 × 10^−8^ mm. Substituting ΔL and ΔL’ into the above Equation (19), we can get that the tilt angle before leveling = 1.12 × 10^−6^°, and the tilt angle after leveling is reduced by 0.71 × 10^−6^°. The simulation results can be obtained as follows: before and after leveling, the tilt angle of the working platform can be reduced to 63%.

## 5. Conclusions

A leveling method based on the inverse piezoelectric properties of piezoelectric materials is proposed to apply PZT materials to the field of horizontal adjustment. (1) The relationship between the point of action of the force and the component force of each leg is deduced through the analysis of the space parallel force system of the drivable legs. (2) Simulation experiments were conducted by using the transient structure module in ANSYS, and the results showed that the theoretical calculated value of the leveling is within the same magnitude as the simulated value, and the horizontal adjustment function can be realized with micron-level accuracy. (3) Through the platform’s flatness verification, when the tilt angle and the accuracy threshold satisfy its geometric relationship, the leveling requirements are met. This leveling method is innovative, and the effect is stable, meeting the high-precision requirements of working platforms and providing a new concept for leveling technology.

## Data Availability

The original contributions presented in the study are included in the article; further inquiries can be directed at the corresponding author.
